# 

**Published:** 1915-12-25

**Authors:** 


### Gifts to Hospitals.

The Queen has sent a Christmas donation of £10 to the Chelsea Hospital for Women.

The Fishmongers' Company has recently made the following, among other, grants: All Saints' Convalescent Hospital, Eastbourne (for Company's bed), £30; Brompton Hospital for Consumption, £31 10s.; City of London Truss Society, £21; Convalescent Home for Poor Children, St. Leonards-on-Sea, £10 10s.; London Hospital, £52 10s.; National Hospital for Diseases of the Heart, £6 6s.; National Truss Society, £15 15s.; Queen Charlotte's Lying-in Hospital, £5 5s.; Royal Dental Hospital, £10 10s.; Royal Free Hospital, £21; Royal National Sanatorium, Bournemouth, £18 18s.; Royal Surgical Aid Society, £21 10s.

### A Telegram as an Heirloom.

Mr. Richard Clement Lucas, F.R.C.S., late senior surgeon to Guy's Hospital, and late Vice-President of the Royal College of Surgeons, in leaving his estate to his son, also bequeathed to him as an heirloom the telegram ordering the retreat from Spion Kop.

### The Late Dr. George A. Heron.

At a meeting of the Council of the London and Counties Medical Protection Society, Limited, held on December 16, 1915, at the offices of the Society, under the presidency of Colonel Bensley, it was unanimously resolved: "That the members of the Council desire to put upon record their very deep sense of the great loss sustained by the Society by the death of its President and Chairman of Council, Dr. George Allan Heron. During the twenty-three years that Dr. Heron was Chairman, he had devoted his valuable time and best energies to the interests of the Society, for which the Council and members cannot fail to be sincerely grateful." Dr. Heron was a very industrious, hard-working, and useful member of the profession, who did much voluntary work for it and the service of the public and medical charities. He will be missed in many fields, but his example has stimulated many of his fellows to follow his example. R.I.P.

RATES OF SUBSCRIPTION: Twelve months, 6/6; Six months, 4/-; Three months, 2/-, post free to any address in the United Kingdom. Abroad, Twelve months, 8/8; Six months, 5/-; Three months, 2/6, post free.—Address The Subscription Dept., "THE HOSPITAL," The Hospital Building, 28/29 Southampton Street, Strand, London, W.C.

28/29 Southampton Street, Strand, London, W C

**Send your Order now**

**FOR THE 1916 EDITION OF**

### Burdett's Hospitals and Charities

**The Year Book of Philanthropy and Hospital Annual.**

**Edited by Sir HENRY BURDETT, K.C.B., K.C.V.O.**

THIS authoritative annual work of reference will be published early in the New Year, and it is advisable to place your order without delay, as owing to the increased costs of production the edition will be limited.

*OF ALL BOOKSELLERS, OR OF*

**THE SCIENTIFIC PRESS, LTD.,**

